# Supplementary material for: Content-rich biological network constructed by mining PubMed abstracts
Source: BMC Bioinformatics. 2004 Oct 8;5:147. doi: 10.1186/1471-2105-5-147 (PMC528731; doi:10.1186/1471-2105-5-147)
Supplement: Additional File 2 — The original results of the above study (non-essential files are deleted to keep the file size under the limit set by BMC bioinformatics). [file 1471-2105-5-147-S2.bz2 › chilibotAdditionalFile2/dip10/501ID7878020E10667/html/TGFBR1_TGFBR2.html]

 


 **TGFBR1** and **TGFBR2** 
  
Found 40 abstracts in PubMed, retrieved 10.  
 

 What does Google say? 
 PDF only 
| .edu only 

---

**Interactive relationship** (e.g. stimulation, inhibition, etc)

**Non-interactive relationship** (e.g. studied together, co-existance, homology, etc.)

- No mutations of ALK5  [ **TGFBR1** ]  or  **TGFBR2**  were found.  Ref: 11408925 Int J Oncol, 2001
- We generated mice expressing an activated TGF beta type I receptor  [ **TGFBR1** ]  or dominant negative TGF beta type II receptor  [ **TGFBR2** ]  under control of the mouse mammary tumor virus promoter.  Ref: 12808151 Proc Natl Acad Sci U S A< MedlineTA>Proc Natl Acad Sci U S A, 2001
- We also analysed ALK5  [ **TGFBR1** ]  and  **TGFBR2**  for mutations, cell surface localisation of  **TGFBR2**  and ENG, and TGFB1 response.  Ref: 11408925 Int J Oncol, 2001
- We therefore studied the expression of UPA, UPAR, IGF2R, ALK5  [ **TGFBR1** ] ,  **TGFBR2** , TGFBR3, ENG, ALK1, TGFB1, TGFB2, and TGFB3 in a series of 14 pancreatic carcinoma cell lines.  Ref: 11408925 Int J Oncol, 2001
- However, we observed a markedly low TGF beta type I receptor  [ **TGFBR1** ]  protein level, while the malignant endometrium showed a significantly higher TGF beta type II receptor  [ **TGFBR2** ]  protein level than the normal endometrium.  Ref: 12213293 Cancer Lett, 2002
- These genes were TGF beta type I receptor  [ **TGFBR1** ]  gene  **TGFBR1** , TGF beta type II receptor  [ **TGFBR2** ]  gene  **TGFBR2** , SMAD2 gene SMAD2, SMAD3 gene SMAD3, SMAD4 gene SMAD4, and SMAD7 gene SMAD7, all of which compose the TGF beta1 signaling pathway.  Ref: 12202987 J Hum Genet, 2002
- These results suggest that the ALK5  [ **TGFBR1** ]  SMAD4 part of the TGFB signalling pathway is a major target for inactivation in pancreatic carcinomas, that the expression of  **TGFBR2** , TGFBR3, and receptors involved in TGFB activation are maintained,.  Ref: 11408925 Int J Oncol, 2001
